# Mechanical Properties of Alkasite Material with Different Curing Modes and Simulated Aging Conditions

**DOI:** 10.3390/ma17112777

**Published:** 2024-06-06

**Authors:** Visnja Negovetic Mandic, Laura Plancak, Danijela Marovic, Zrinka Tarle, Milena Trutina Gavran, Matej Par

**Affiliations:** 1 Department of Endodontics and Restorative Dentistry, School of Dental Medicine, University of Zagreb, Gunduliceva 5, 10000 Zagreb, Croatia; vnegovetic@sfzg.hr (V.N.M.); marovic@sfzg.hr (D.M.); tarle@sfzg.hr (Z.T.); 2 School of Dental Medicine, University of Zagreb, Gunduliceva 5, 10000 Zagreb, Croatia; lauraplancak1234@gmail.com; 3 Department of Morphology and Anthropology, Study of Dental Medicine, School of Medicine, University of Mostar, Zrinskog Frankopana 34, 88000 Mostar, Bosnia and Herzegovina; mtrutina1@gmail.com

**Keywords:** dental materials, alkasite, resin composite, flexural properties, crosslink density

## Abstract

This study aimed to evaluate the micro-mechanical and macro-mechanical properties of self-cured and light-cured alkasite and to investigate how accelerated degradation in acidic, alkaline, and ethanol solutions affects the macro-mechanical properties of self-cured and light-cured alkasite. The specimens of the alkasite material (Cention Forte, Ivoclar Vivadent) were prepared according to the following three curing modes: (1) light-cured immediately, (2) light-cured after a 5-min delay, and (3) self-cured. Microhardness was tested before and after immersion in absolute ethanol to indirectly determine crosslink density, while flexural strength and flexural modulus were measured using a three-point bending test after accelerated aging in the following solutions: (1) lactic acid solution (pH = 4.0), (2) NaOH solution (pH = 13.0), (3) phosphate-buffered saline solution (pH = 7.4), and (4) 75% ethanol solution. The data were statistically analyzed using a two-way ANOVA and Tukey post hoc test. The results showed that the microhardness, flexural strength, and flexural modulus were significantly lower in self-cured specimens compared to light-cured specimens. A 5-min delay between the extrusion of the material from the capsule and light curing had no significant effect on any of the measured properties. A significant effect of the accelerated aging solutions on macro-mechanical properties was observed, with ethanol and alkaline solutions having a particularly detrimental effect. In conclusion, light curing was preferable to self-curing, as it resulted in significantly better micro- and macro-mechanical properties, while a 5-min delay between mixing the capsule and light curing had no negative effects.

## 1. Introduction

Resin composites are one of the most commonly used materials in restorative dentistry [1,2]. Despite the many advantages of these materials, such as ease of handling, good mechanical properties, and esthetics, the main obstacle to the longevity of composite restorations is marginal microleakage and secondary caries [3]. A possible solution to overcome the development of secondary caries can be found in the use of ion-releasing, acid-neutralizing composites. An improved version of one such material, called “alkasite”, has recently become commercially available. This material contains reactive glasses [4], in addition to conventional reinforcing fillers, which provide ion release, acid neutralization, and satisfactory mechanical properties for restorative applications. The alkasite was initially launched as a powder/liquid material that was dispensed and mixed by hand (marketed as Cention N) and has more recently become available in capsules (marketed as Cention and Cention Forte). Some compositional differences among these material versions are likely but have not been explicitly stated by the manufacturer. Regardless of the material version, there is a reasonable amount of evidence for the good performance of the alkasite under in vitro conditions [5,6,7,8,9,10,11,12], as well as in randomized clinical trials [13,14,15,16,17], as summarized below.

In vitro studies have shown that alkasite can release calcium, phosphate, and fluoride ions [5,18] to improve the mineral content of artificially demineralized dentin [6] and maintain the mineral content of enamel [7] and dentin [8] under acidic conditions. Also, the elevation of the pH of the *S. mutans* biofilm [9] and the remineralization of artificial proximal enamel caries were shown in vitro [10]. The alkasite showed better marginal adaptation than a conventional nanohybrid composite after thermomechanical loading in a chewing simulator [11]. Although the alkasite showed lower bond strength than conventional composites and compomers, its bond strength was similar to that of resin-modified glass ionomer cements [12].

Considering that the material is relatively new, only short-term results of clinical studies with a duration of up to 2 years are published. Despite the lower marginal integrity of alkasite compared to a conventional bulk-fill composite in a randomized clinical trial (RCT) with a 2-year follow-up, no secondary caries was observed, and the overall clinical performance of alkasite was found to be similar to that of the conventional bulk-fill composite [13]. In an RCT on non-carious cervical lesions, the alkasite showed better retention, marginal integrity, anatomic form, and color stability than a resin-modified glass ionomer after 1 year [14]. Other RCTs reported that the alkasite used to restore Class II restorations in deciduous molars performed similarly to a glass ionomer cement, according to the standardized FDI criteria after 1 year [15], as well as similar performance to a nanofilled composite in permanent molars in children after one year [16]. In Class II restorations evaluated according to the United States Public Health Service (USPHS) criteria, after 1 year, the alkasite showed similar performance to a conventional composite [17].

Most of the available evidence for the good performance of the alkasite materials has been collected for the light-cured material. As the manufacturer states that the alkasite is “a self-curing material with light-curing option”, the behavior of the self-cured material is relevant. There is evidence that the alkasite performs less well with self-curing than with light curing in terms of several properties, namely the degree of conversion, flexural strength (FS), flexural modulus (FM), and water sorption and solubility [19]. Hence, the present study aimed to evaluate the micro-mechanical (microhardness and susceptibility to ethanol softening) and macro-mechanical properties (FS and FM) of self-cured and light-cured alkasite. Since the macro-mechanical properties of alkasite have been shown to be pH-dependent [20], the second aim of the present study was to evaluate how aging in solutions with different pH values affects the FS and FM of self-cured and light-cured alkasite. In addition, immersion in ethanol was used as an alternative means of accelerated material aging [21]. The null hypotheses assumed that the curing conditions would have no effect on (1) baseline microhardness (MH), (2) MH after immersion in ethanol, (3) FS, and (4) FM. To investigate the effect of the immersion medium on the macro-mechanical properties, an additional null hypothesis assumed that (5) FS and FM would not be affected by immersion in four different media, namely a neutral solution, an alkaline solution, an acidic solution, and a 75% ethanol solution.

## 2. Materials and Methods

### 2.1. Specimen Preparation

The latest version of the alkasite material, Cention Forte (Ivoclar Vivadent, Schaan, Liechtenstein), was examined. According to the manufacturer’s instructions, the Cention Forte capsule was activated by pressing the plunger onto a flat surface and inserted into a capsule mixer. After mixing according to the manufacturer’s instructions (15 s at a room temperature of 22–24 °C), the material was extruded into custom-made Teflon molds, covered with polyester strips on both sides of the molds and pressed with a thick glass plate to remove excess material. The test specimens were produced at a temperature of 22 ± 1 °C.

Cylindrical specimens (d = 6 mm; h = 2 mm or 4 mm) were prepared for the MH measurements [22], while rod-shaped specimens (16 mm × 2 mm × 2 mm) were used for the FS and FM tests [20], as described in detail below.

### 2.2. Microhardness Measurements

The specimens were prepared in three groups (*n* = 8 per group) according to the following curing modes:

1. Light-cured immediately,
2. Light-cured after a 5-min delay,
3. Self-cured (i.e., undisturbed in the dark for 20 min).

For each group, a set of 2 mm thick specimens and another set of 4 mm thick specimens were prepared to evaluate the effects of layer thickness on MH and ethanol softening.

For light curing in the 1st and 2nd groups, specimens were illuminated from the “top” surface for 20 s with an LED curing unit Bluephase PowerCure (Ivoclar Vivadent, Schaan, Liechtenstein) with a radiant exitance of 1050 mW/cm^2^. This radiant exitance was checked with a NIST-referenced UV-vis spectrophotometer system (MARC; BlueLight Analytics, Halifax, NS, Canada). The cured specimens were stored in black containers in distilled water in a laboratory incubator at 37 °C. To remove the resin-rich layer and obtain a high-gloss surface for the MH measurements, specimens were ground with a P4000 silicon carbide paper under running water and then polished using a 0.05 µm Al_2_O_3_ suspension [23]. The Vickers MH was measured on the specimen surfaces (0 mm), 2 mm, and 4 mm, using a microhardness tester (CSV-10; ESI Prüftechnik GmbH, Wendlingen, Germany) with a load of 100 g and dwell time of 15 s. The Vickers MH was automatically calculated by the software according to the equation VH = 0.1891 × F/d^2^, where VH = Vickers hardness, F = load (N), and d = diagonal length of indentation (mm). Five repeated measurements were performed on the central part of each sample. The mean of the five measurements was calculated and treated as a statistical unit [23].

After the baseline MH measurements, the specimens were immersed in absolute ethanol for 7 days, and the Vickers MH was measured again using the same parameters. The ratio of MH values after/before immersion in ethanol was evaluated as an indicator of crosslink density [22].

### 2.3. Flexural Strength and Modulus Measurements

The specimens for FS and FM measurements were prepared according to the same three curing modes as described above for the MH measurements.

For light curing in the 1st and 2nd groups, the specimens were illuminated with an LED curing unit Bluephase PowerCure (Ivoclar Vivadent, Schaan, Liechtenstein) with radiant exitance of 1050 mW/cm^2^ in three consecutive curing cycles of 20 s each. The central part of the specimen was illuminated first, followed by two illuminations at both ends of the specimen [24]. The same light-curing protocol was repeated on the other side of the specimen. The cured specimens (either light-cured or self-cured) were removed from the molds, excess material was removed with a sharp hand instrument, and then the specimen edges were finished using silicon carbide paper of P2000 roughness.

Each of the three groups was sub-divided according to the following immersion media:

1. Lactic acid solution (pH = 4.0),
2. 1 M NaOH solution (pH = 13.0),
3. Phosphate-buffered saline solution (pH = 7.4),
4. 75% ethanol solution.

This study design resulted in 12 experimental groups (3 curing conditions x 4 immersion media), with *n* = 20 in each group. Each group of specimens was individually stored in a closed Eppendorf tube filled with 15 mL of the respective immersion solution and stored at 37 °C in a laboratory incubator for 15 days. The immersion solution was replaced every 5 days. After completion of artificial aging, the specimens were loaded in a universal testing machine (Inspekt Duo, Hegewald & Peschke, Nossen, Germany) immersed in distilled water at room temperature until fracture. A three-point bending fixture with an inter-support span of 12 mm and crosshead speed of 0.5 mm/min according to NIST 4877 was used [25]. The FS and FM were calculated according to the following equations:

$$FS=\frac{3F_{f}l}{2bh^{2}}$$

$$FM=\frac{F_{l}l^{3}}{4bh^{3}y_{l}}$$

where $\;F_{f}$ = force at fracture (N), l = span between supports (mm), b = specimen width (mm), h = specimen height (mm), $F_{l}$ = force at the end of the linear part of the force–deflection diagram (N), and $y_{l}$ = deflection at the end of the linear part of the force–deflection diagram (mm) [25].

### 2.4. Statistical Analysis

After checking the normal distribution using normal Q-Q plots, the MH data were analyzed using a two-way ANOVA with the factors “layer thickness” and “curing mode”, while the FS and FM data were analyzed using a two-way ANOVA with the factors “immersion medium” and “curing mode”. As statistically significant interactions were observed between the factors, the analysis was followed by one-way ANOVAs to determine the effects of each factor. The *p*-values were adjusted for multiple comparisons using Tukey post hoc test. Rather than presenting individual *p*-values for each of the pairwise comparisons, the results of the statistical analysis were summarized by reporting the means as statistically similar or dissimilar at the 0.05 level of significance. Statistical analysis was performed using the SPSS v25.0 software package (IBM, Armonk, NY, USA).

To evaluate the material reliability, a Weibull analysis was performed using the function ln ln [1/(1 − Pf)] = m (ln σ − ln σθ), where Pf = probability of failure, m = Weibull modulus, σ = flexural strength at failure, and σθ = characteristic strength [26]. In this equation, the parameter “m” is the shape parameter of the Weibull distribution, which can be used to quantify material reliability. Weibull graphs were plotted using 20 data points for the *n* = 20 per experimental group, and a linear function was fitted to the scatterplot. Weibull analysis was performed using OriginPro (version 9.1; OriginLab, Northampton, MS, USA).

## 3. Results

**Figure 1:**
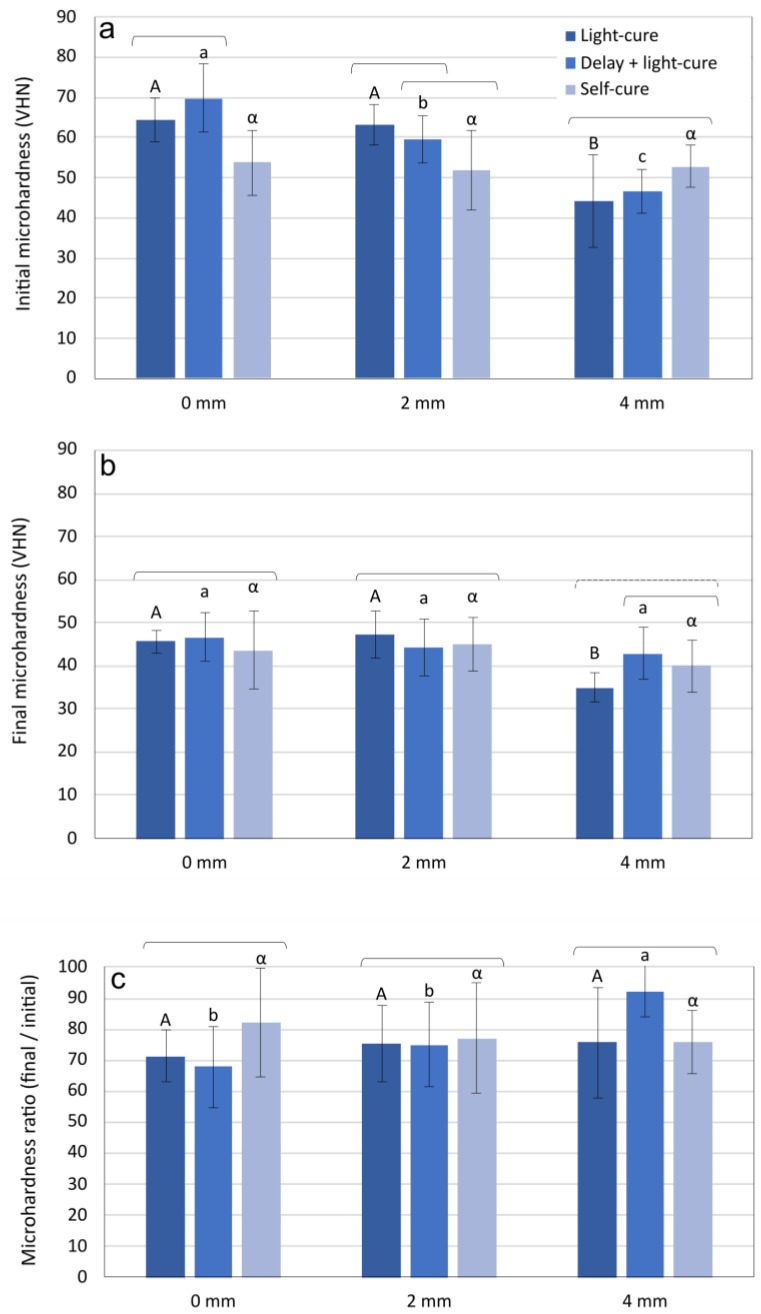
Mean values and standard deviations of initial microhardness (**a**), final microhardness (**b**), and the ratio between final and initial microhardness (**c**). Statistically similar values (*p* > 0.05) for comparisons among layer thicknesses are marked with the same uppercase letters (light-cured specimens), lowercase letters (delayed light-cured specimens), and Greek letters (self-cured specimens). Square brackets indicate statistically similar values (*p* > 0.05) for comparisons among different curing modes within each layer thickness.

For the 0 mm and 2 mm thicknesses, baseline MH values (before immersion in ethanol) were significantly lower for the self-cured specimens than for the light-cured specimens (*p* = 0.001–0.025, Figure 1a). In contrast, the 4 mm layer thickness showed statistically similar MH values (*p* = 0.161–0.843) for all curing modes. After ethanol immersion, the MH values at 0 mm and 2 mm were statistically similar for all curing conditions (*p* = 0.621–0.631), while at 4 mm, delayed curing resulted in significantly higher MH values than immediate curing (*p* = 0.017, Figure 1b. The final/initial MH ratios (calculated by dividing the values after ethanol immersion by the values before immersion) were statistically similar within each layer thickness regardless of the curing mode (*p* = 0.064–0.963, Figure 1c).

**Figure 2:**
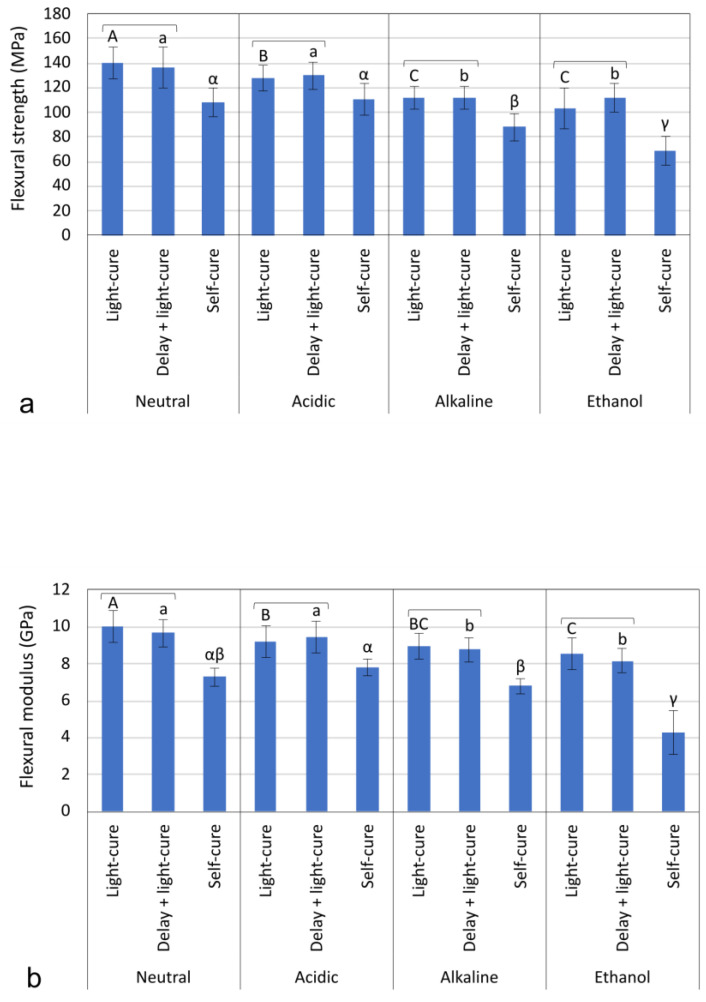
Mean values and standard deviations of flexural strength (**a**) and flexural modulus (**b**). Statistically similar values (*p* > 0.05) for comparisons among immersion media are marked with same uppercase letters (light-cured specimens), lowercase letters (delayed light-cured specimens), and Greek letters (self-cured specimens). Square brackets indicate statistically similar values (*p* > 0.05) for comparisons among different curing modes within each immersion medium.

The results of FS (Figure 2a) and FM (Figure 2b) show no statistically significant difference between immediate and delayed light curing, regardless of the immersion medium (*p* = 0.113–0.998). In contrast, the self-cured specimens showed significantly lower FS and FM values compared to the light-cured specimens (without or with delay) in all immersion media (*p* < 0.001). Statistically significant differences were found when comparing the FS and FM values among the immersion media (*p* < 0.001) with the following general rankings: neutral > acidic > alkaline > ethanol. This order of reduction in FS and FM was observed regardless of the curing mode. The threshold value of 80 MPa defined in ISO 4049 [25] as “minimum acceptable FS” was exceeded in all light-cured groups (without or with delay) regardless of the immersion medium. Only in the self-cured group immersed in ethanol was the FS below the 80 MPa required by ISO 4049 [25].

**Figure 3:**
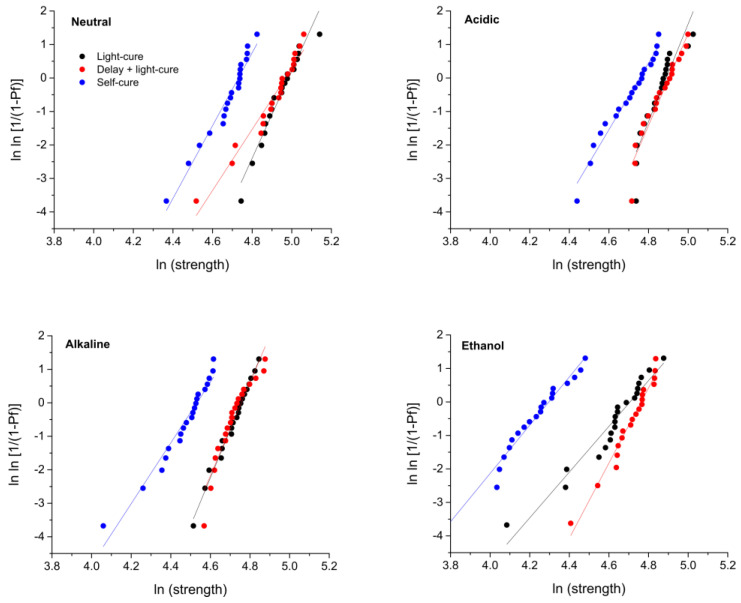
Weibull plots in which material reliability is represented as slopes of fit lines.

The Weibull diagrams in Figure 3 show how the log-transformed FS values are distributed according to the probability of failure. A higher reliability is indicated by a steeper fit line. The Weibull modulus values (i.e., the slopes of the fit lines) are shown in Table 1. The statistical significance of the differences in the estimated Weibull moduli can be judged by observing the lack of overlap of their 95% confidence intervals. Within each of the immersion solutions, the Weibull moduli did not differ significantly among the curing modes. In general, the experimental group immersed in ethanol showed the lowest reliability, but without statistical significance due to imprecise estimates, i.e., wide 95% confidence intervals.

## 4. Discussion

This study showed that the micro-mechanical properties (MH) and macro-mechanical properties (FS and FM) of the alkasite material were significantly inferior in self-cured specimens compared to light-cured specimens. On the other hand, a 5-min delay between material extrusion from the capsule and light curing (simulating prolonged material handling, placement and shaping of the restoration) had no significant effect on any of the measured properties. Hence, null hypotheses 1–4 were rejected. Null hypothesis 5 was also rejected, as a significant effect of the immersion media on macro-mechanical properties was observed, with ethanol and alkaline solutions having a particularly detrimental effect.

The depth-dependent curing efficiency was evaluated by measuring the MH at layer thicknesses of 0 mm, 2 mm, and 4 mm. Although the alkasite is a dual-cured composite, it could theoretically be cured to an unlimited layer thickness, as the chemically initiated polymerization should compensate for the lack of light exposure in thicker layers [27]. However, by using MH measurements as an indirect indicator of curing efficiency [28], our study showed a decrease in MH with increasing layer thicknesses, indicating that the heterogeneity of the cure through depth persists despite the alkasite’s self-curing ability. These results are consistent with our results for FS and FM, which are significantly lower for self-cured specimens. The practical implications of lower cure at deeper layers are insufficient mechanical properties, as well as biocompatibility concerns due to a higher amount of leachable monomers, which may have toxic effects when placed on highly permeable dentin close to the pulp [29].

The results for the initial MH values indicate that self-curing at thicknesses of 0 mm and 2 mm resulted in poorer micro-mechanical properties than light curing, while the self-cured specimens at 4 mm showed no difference in MH values to the light-cured specimens. The latter is due to the fact that light penetrates poorly to the bottom of a 4 mm thick layer; therefore, this part of the specimen was effectively left to self-cure. Another finding from the initial MH data is that the 5-min delay before light curing had no effect on the curing efficiency. The lack of a negative effect of the 5-min delay was confirmed by the FS and FM data, which showed statistically similar values for the immediately light-cured group and the group that was light-cured with a delay.

The final MH results (after immersion in ethanol) were mostly leveled to statistically similar values for all curing modes. The microhardness ratio, which indicates the crosslinking density of the polymer network [30], also appears to be unaffected by the curing modes. The comparisons among layer thicknesses also showed statistically similar MH ratios, with the exception of the delayed light curing at 4 mm, which showed a significantly higher MH ratio compared to the other two thicknesses. This result is most likely due to the fact that the final MH values of the delayed self-cured group at 4 mm were significantly higher than those of the other two groups. This is likely due to the fact that the delay allowed for some degree of mobility before the polymer network was immobilized by light curing, and ultimately resulted in a slightly higher conversion than the immediately light-cured group. Nevertheless, it cannot be ruled out that the lack of statistically significant effects was caused by high coefficients of variability (i.e., relative standard deviations) for the MH ratio data, which ranged from 9.1 to 23.5%.

In general, the crosslinking density of the methacrylate polymer network is influenced by the number of free radicals present in a unit volume during polymerization. Since the free radicals act as growth centers of the polymer chains, higher initiation rates lead to a more crosslinked network, while lower initiation rates and fewer free radicals per unit volume lead to more linear polymer chains [31]. The structure of the polymer network (crosslinked vs. linear) influences its tendency to soften when permeated by a suitable solvent [32]. Hence, the evaluation of MH values before and after ethanol immersion, usually expressed as the final/initial MH ratio, is considered an indirect measure of crosslinking density. Although the finding that the MH ratios are statistically similar for all curing modes indicates a similar crosslink density of the resulting polymer networks, a practically more important finding is that the initial MH values clearly reflect poorer curing in the self-cured group than in the two light-cured groups. Hence, our MH results considered overall indicate that the alkasite should be light-cured to maximize its micro-mechanical properties and that a depth-dependent decrease in cure should be expected regardless of the curing approach used. The recommendation to prefer light curing over self-curing of the alkasite material was also mentioned in a recent study on its mechanical properties, water sorption, and solubility [19].

There is only one published study on ethanol softening of an early version of the alkasite material (Cention N) in which the Knoop hardness was compared after and before ethanol immersion to indirectly assess the crosslink density [33]. In that study, only the top surface of the light-cured specimens was evaluated, and the reported MH ratio was 48%, which was considerably lower compared to the MH ratios of the light-cured specimens obtained in our study (72–76%). Considering the methodological differences and the unknown material modifications that occurred during the development of Cention N, tested in the aforementioned study [33], to Cention Forte, which was tested in our study, it is not possible to discuss these differences in more detail.

In agreement with the micro-mechanical results (MH), the macro-mechanical results (FS and FM) showed a significantly poorer performance of the self-cured group compared to the two light-cured groups, between which there was no significant difference. These results generally indicate that (I) the alkasite should be light-cured to achieve optimal micro- and macro-mechanical properties, and (II) that a delay of up to 5 min before light curing does not significantly affect the tested properties. Immersion in different aggressive media was used to detect the possible differences between curing modes that may not be observed under neutral conditions [34]. This so-called “accelerated aging” by immersing the specimens in different solutions is assumed to accelerate the degradation at specific sites of methacrylate-based composites; the 75% ethanol and alkaline solution accelerate the hydrolysis of the methacrylate polymer and the silane-mediated resin/filler interface [34], while the acidic solution should accelerate the degradation of the soluble glass, which becomes more soluble at lower pH values [35]. The aforementioned sites targeted by the different solutions for accelerated material degradation are theoretical in nature and are not mutually exclusive, as the degradation process is complex and influenced by an interaction of multiple factors [36]. For example, hydrolysis is facilitated by higher concentrations of OH^−^ ions in the solution but also depends on the pH of the environment, the ability of the solvent to penetrate the material, the elution of material components, and the swelling of the polymer network, leading to a higher diffusivity of the solution and, thus, increase the degradation rate. The degradation processes of the individual components of the composite material cannot be observed separately but only as an integrated response of the entire material to exposure to an aggressive solution used for accelerated aging.

In view of this discussion, the solutions used for accelerated aging in the present study were selected as suggested in previously published studies, namely the acidic solution with a pH of 4.0 [27,37], the alkaline solution with a pH of 13 [34], and 75% ethanol [38]. The most severe deterioration in macro-mechanical properties was observed for the self-cured specimens immersed in ethanol, with FS values 17.9–36.4% lower and FM values 14.7–41.0% lower than the corresponding values measured in the neutral solution. This can be explained by the easy diffusion of ethanol through the incompletely polymerized resin (as shown by the lower MH values of the self-cured specimens) and its tendency to cause hydrolysis both within the resin and at the silane interface between resin and fillers [34]. In addition, ethanol is characterized by a similar Hoy solubility parameter to dimethacrylate resins [39], allowing it to rapidly penetrate and expand the polymer network, especially when incompletely cured, as was the case for the self-cured alkasite specimens in our study. Elution of unreacted monomers facilitates the penetration of ethanol into the incompletely cured resin, further enhancing degradation [40]. The second highest degradation effect was observed for the alkaline solution, with FS values 18.7–20.0% lower and FM values 6.8–10.5% than those measured in the neutral solution. While the neutral solution expectedly caused the less extensive degradation, it is interesting to note that the acidic solution reduced FS values by only 4.8–8.7% and FM values by 2.2–7.8% compared to the neutral solution. This result indicates the ability of alkasite to maintain its mechanical properties at a low pH, despite the fact that part of its filler (i.e., the reactive glasses) dissolves at a faster rate [4]. The pH-dependent dissolution of these glasses is potentially advantageous for clinical use, as the release of remineralizing ions and alkalization can be self-regulated according to intermittent bacterial acid production [7].

A reliability analysis using Weibull statistics was performed as an additional means of evaluating the FS data [26], as it is able to reveal differences in material behavior that may go unnoticed in a conventional analysis of variance. Although the ethanol group showed the least reliability compared to the other groups, which is consistent with the generally poorer performance in ethanol, no significant differences in material reliability were found among curing modes. The low discriminatory power of the Weibull analysis in the present study can be attributed to inaccurate parameter estimates caused by deviations of the data from the fit lines in the Weibull plots. These deviations indicate the possible presence of different subpopulations of critical flaws within the specimens that had their own Weibull distributions and therefore did not fit well with the general model that assumes a single population of flaws that follow a single Weibull distribution. With the current sample size of *n* = 20 per experimental group, these subpopulations of flaws could not be distinguished.

Although, according to the manufacturer’s instructions, Cention Forte should self-cure after 6.5 min from mixing [41], in our study, the material appeared rubbery and incompletely set after this time; hence, a longer time of 20 min was selected for the self-curing mode. Even after 20 min of self-curing, the specimen’s surface was still sticky and appeared incompletely cured. Hence, light curing of the alkasite seems to be the only viable method for clinical use, as it is impractical to wait 20 min or longer for the material to self-cure.

Although the alkasite degraded to varying degrees in the acidic, alkaline, and ethanol solutions, it met the minimum acceptable FS of 80 MPa specified by the ISO [42] for all light-cured specimens. It should be noted that the above requirement described in the ISO 4049 [25] protocol for resin-based composites is defined for short-term aging of one day in distilled water at 37 °C, while the degradation effects were considerably exaggerated by the more aggressive solutions used in our study. The ability of alkasite to meet the ISO requirement for minimum FS even under such unfavorable conditions indicates its optimal mechanical stability and potential for use in load-bearing areas. This is consistent with reports of alkasite’s good performance under clinical conditions, albeit with short follow-up times of only 1–2 years [13,14,15,16,17].

Our results of inferior mechanical properties in self-cured specimens are consistent with the usual behavior of dual-curing resin composites, which are known to achieve lower cure when self-cured than when light-cured [43,44,45]. This was also confirmed by two earlier studies on Cention [19,46]. However, it appears that the early iteration of the alkasite material (hand-mixed version, Cention N) was better able to achieve optimal polymerization when self-cured. This is supported by an early study on curing kinetics, which shows that the same degree of conversion can be achieved regardless of the curing mechanism [27]. Additionally, a study on the mechanical properties of Cention N showed statistically similar FS [47] for light-cured and self-cured specimens, while another study even showed significantly higher FS for self-cured specimens [48]. In the latter two studies, the FS of Cention N (after storage at neutral pH) amounted to 83–86 MPa [47] and 62–83 MPa [48], which is much lower than the FS values reported for the newer encapsulated version (Cention), i.e., above 100 MPa [19], and the FS values of the latest version (Cention Forte) in the present study (108–140 MPa). Although the compositional modifications made during the development of the alkasite have not been disclosed by the manufacturer, the modifications to improve flexural strength usually involve an increase in the amount of filler and/or modifications in particle size geometry, generally contributing to reduced mobility of the resin monomers at the micro-scale, ultimately diminishing the efficiency of curing [49]. Hence, it is possible that these modifications have reduced the ability of the self-curing mechanism in newer versions (Cention and Cention Forte), so that the self-cured specimens had a lower degree of conversion [46] and, consequently, lower mechanical properties [19] than light-cured specimens. On the other hand, there are also reports of high FS values even for the early version of the alkasite (Cention N) in the range of 100–120 MPa [27,33], suggesting that the differences in the measured values may be due to methodological particularities rather than some concrete trends in the improvement of the mechanical properties of alkasite due to the development of newer versions. The inconsistencies in the results for macro-mechanical properties for different “generations” of the alkasite material are compounded by a study showing that the FS and FM of Cention N were significantly higher for the self-cured mode than for the light-cured mode [50]. These discrepancies may be partly attributed to (I) the variations in the behavior of the hand-mixed material, and (II) the specific instructions for use for Cention Forte that recommend different mixing times depending on the ambient temperature. The former suggests that it is impossible to fully standardize specimen preparation, and the latter indicates a high technical sensitivity of the mixing process, which opens up the possibility of introducing additional variability in the curing behavior of the material.

The general limitation of any study on commercial materials is related to the limited and superficial knowledge of the composition of the material, as this information is usually not shared by the manufacturer. Therefore, all degradation processes can only be observed at the level of the whole material, while the attribution of certain behaviors to specific material components necessarily remains speculative. This applies in particular to the alkasite material, the composition of which has changed to an indeterminate extent in the course of the development of Cention N and Cention up to the latest version, Cention Forte. One should also mention the limitations of the present study design, which uses exposure to a strongly acidic and a strongly alkaline solution for prolonged periods and prolonged exposure to ethanol to highlight the effects of the different degradation processes but does not correspond to the conditions realistically encountered in the oral cavity.

## 5. Conclusions

Within the limitations of this in vitro study on the micro- and macro-mechanical properties of the alkasite material Cention Forte cured under different conditions, the following can be stated:

1. Light curing is preferable to self-curing, as it results in significantly better microhardness, flexural strength, and flexural modulus;
2. A 5-min delay between mixing the capsule and light curing had no negative effect on the aforementioned properties;
3. After immersion in various solutions which enhanced material degradation, the significantly lowest flexural strength and flexural modulus were consistently observed in the self-cured specimens.

## Figures and Tables

**Table 1:** Mean values and boundaries of 95% confidence intervals for Weibull modulus.

		Weibull Modulus		
Immersion Medium	Curing Mode	Mean Value	95% Confidence Interval	
			Lower Bound	Upper Bound
**Neutral**	Light cure	13.06	7.03	19.09
	Delay + light cure	9.12	4.97	13.27
	Self-cure	10.87	6.18	15.57
**Acidic**	Light cure	14.57	2.38	26.76
	Delay + light cure	13.73	6.20	21.27
	Self-cure	9.96	6.39	13.54
**Alkaline**	Light cure	14.29	11.15	17.43
	Delay + light cure	13.94	4.79	23.10
	Self-cure	9.08	5.06	13.10
**Ethanol**	Light cure	6.83	3.20	10.47
	Delay + light-cure	11.23	6.83	15.63
	Self-cure	7.21	4.68	9.74

## Data Availability

The original contributions presented in the study are included in the article, further inquiries can be directed to the corresponding author.
